# Assessing the Quick Inventory of Depressive Symptomatology Self-Report scores to predict continuous employment in mood disorder patients

**DOI:** 10.3389/fpsyt.2024.1321611

**Published:** 2024-04-17

**Authors:** Yasuyuki Matsumoto, Hitoshi Sakurai, Yumi Aoki, Yoshikazu Takaesu, Isa Okajima, Hisateru Tachimori, Masami Murao, Taku Maruki, Takashi Tsuboi, Koichiro Watanabe

**Affiliations:** ^1^ Department of Neuropsychiatry, Kyorin University Faculty of Medicine, Tokyo, Japan; ^2^ Psychiatric and Mental Health Nursing, Graduate School of Nursing Science, St. Luke’s International University, Okinawa, Tokyo, Japan; ^3^ Department of Neuropsychiatry, Graduate School of Medicine, University of the Ryukyus, Okinawa, Japan; ^4^ Department of Psychological Counseling, Faculty of Humanities, Tokyo Kasei University, Tokyo, Japan; ^5^ Endowed Course for Health System Innovation, Keio University School of Medicine, Tokyo, Japan

**Keywords:** absenteeism, bipolar, depression, employment, QIDS-SR, severity

## Abstract

**Objective:**

Depression significantly impacts the job performance and attendance of workers, leading to increased absenteeism. Predicting occupational engagement for individuals with depression is of paramount importance. This study aims to determine the cut-off score which predicts continuous employment for patients with mood disorders using the Quick Inventory of Depressive Symptomatology, Self-Report (QIDS-SR).

**Methods:**

In a prospective observational trial conducted in Tokyo, 111 outpatients diagnosed with either major depressive disorder or bipolar depression were enrolled. Their employment statuses of these participants were tracked over a six-month period after their QIDS-SR scores were recorded. Based on their employment trajectories, participants were categorized into either continuous or non-continuous employment groups. Binary logistic regression was applied to examine the relationship between the QIDS-SR scores and employment outcomes, with adjustments for age, gender, and psychiatric diagnoses. Receiver operating characteristic curves were utilized to identify the optimal QIDS-SR cut-off values for predicting continuous employment.

**Findings:**

Binary logistic regression demonstrated that a lower score on the QIDS-SR was linked to an elevated likelihood of continuous employment (adjusted odds ratio 1.15, 95% CI: 1.06-1.26, p=0.001). The optimal cut-off point, determined by the Youden Index, was 10/11, showcasing a 63% sensitivity and 71% specificity.

**Conclusion:**

The results emphasize the potential of the QIDS-SR as a prognostic instrument for predicting employment outcomes among individuals with depressive disorders. These findings further underscore the importance of managing depressive symptoms to mild or lower intensities to ensure ongoing employment.

## 1 Introduction

Mood disorders, with a particular emphasis on depression, exert a significant influence on job performance and participation, leading to substantial levels of workforce presenteeism and absenteeism (1–3). Often, individuals who rejoin the workforce following a depression-induced sick leave encounter lingering impairments and are frequently prone to repeated periods of absence (4). In light of this, the economic footprint of depression cannot be ignored: around 200 million workdays are forfeited annually, placing a monetary strain between $17 billion and $44 billion on United States employers alone (5). This striking reality underscores the wide-reaching and enduring effects of depression, exemplifying a societal challenge that extends far beyond the personal affliction of the individual (6, 7).

It was estimated that absenteeism due to major depressive disorder (MDD) accounted for 11.5% of the societal economic burden of this illness (8). Therefore, predicting occupational engagement in a timely manner is crucial for devising appropriate management strategies for individuals affected by depression. Various factors, including duration of the ongoing depressive episode, presence of concurrent mental or physical disorders, older age, and a history of previous sick leave, have been identified as influencing work participation among those with depressive symptoms (1). A meta-analysis of 15 prospective cohort studies revealed that the severity of depressive symptoms, based on interviews or self-reports among the working population, was associated with current or future sick leave, with an overall risk ratio of 1.52 (9). Most of these studies, however, included the general working population, rather than patients diagnosed with depressive disorders (10–15). There have been only two studies that examined the effect of symptom severity using the rating scale on sick leave or absenteeism in patients with major depression. In a cohort study of 269 outpatients and inpatients with major depression, individuals on sick leave demonstrated a lower baseline score on the Hamilton Depression Rating Scale (HDRS) compared to those actively working by a paired t-test (16). In a separate cross-sectional study involving 335 employed outpatients with major depression, the factor most closely linked to absenteeism was the severity of depressive symptoms as measured by the HDRS with an adjusted odds ratio of 44 (17). Both studies demonstrated that symptom severity assessed on the HDRS was related to absenteeism. Nonetheless, no study has investigated a clear cut-off value on rating scales in patients with major depression to predict continuous employment.

The Quick Inventory of Depressive Symptomatology, Self-Report (QIDS-SR), a 16-item brief self-report rating scale, evaluates nine depressive symptom domains defined according to the Diagnostic and Statistical Manual of Mental Disorders, Fifth Edition (DSM-5) (18). It is frequently utilized in clinical trials as exemplified by its use in the Sequenced Treatment Alternatives to Relieve Depression trial which involved over 4,000 outpatients with MDD (19). The QIDS-SR also serves as a streamlined assessment tool that requires less time for evaluation compared to the HDRS, thereby enhancing its clinical utility.

The primary objective of the present study is therefore to propose a clinically relevant cut-off score for this scale to predict potential unemployment or sick leave in patients with mood disorders.

## 2 Methods

### 2.1 Design and setting

The study is a prospective observational trial conducted between January, 2021 and December, 2021 at seven psychiatric outpatient services in Tokyo, Japan. The institutional review board at Kyorin University in Tokyo granted approval for this trial (786–01). All participants were provided with a comprehensive explanation of the study and provided written informed consent prior to their participation.

### 2.2 Participants

The study enrolled individuals who fulfilled the following criteria for inclusion: (1) outpatients aged between 20 and 65 years old, (2) diagnosed with either MDD or bipolar disorder according to the DSM-5, and (3) either currently employed, on sick leave, or actively seeking employment. Those who exhibited manic or psychotic symptoms, had a substance use disorder involving alcohol or any drugs, suffered from dementia, serious physical illness, or had suicidal thoughts were excluded from the study.

### 2.3 Procedures

The study participants were assessed using the QIDS-SR at baseline, after which their employment status was monitored for six months. Based on their employment status during the follow-up period, participants were categorized into two groups: the continuous employment group and the non-continuous employment group. The continuous employment group comprised individuals who remained employed throughout the follow-up period whereas the non-continuous employment group included those who experienced unemployment or took sick leave during this time. Sick leave was designated by an official medical certificate attesting to the individual’s inability to work. Concerning part-time workers, those with continuous contracts and regular work patterns were identified as the continuous employment. In contrast, patients with one-off contracts were classified into non-continuous employment group.

### 2.4 Rating scale

The QIDS-SR is a widely recognized self-report questionnaire designed to evaluate the severity of depressive symptoms (20). It examines nine symptom domains, each graded on a scale from 0 (no problem) to 3 (severe problem) based on the individual’s experiences over the preceding week, with higher scores indicating more severe symptoms. The total score, ranging from 0 to 27, helps categorize depression severity as follows: no depression (0-5), mild depression (6-10), moderate depression (11-15), severe depression (16-20), and very severe depression (21 and above). The Japanese version of the QIDS-SR, which was used in the present study, has demonstrated high internal consistency (Cronbach’s α = 0.86), and sufficient correlation with the HDRS and the Beck Depression Inventory (r = 0.67; p < 0.001 and r = 0.86; p < 0.001) (21).

### 2.5 Data analysis

Binary logistic regression was employed to investigate the association between the continuous employment and the QIDS-SR score after controlling for age, gender, and psychiatric diagnoses. To examine the impact of individual depressive symptoms on continuous employment, another binary logistic regression that included the QIDS-SR individual items was conducted after controlling for age, gender, and psychiatric diagnosis. In this analysis, scores of two or higher on individual items were deemed indicative of symptom presence. To assess the fit of the binary logistic regression model, the Hosmer-Lemeshow goodness-of-fit test was conducted. A p-value of less than 0.05 was considered statistically significant. Receiver operating characteristic (ROC) curves analysis was utilized to establish optimal cut-off value for continuous employment based on the QIDS-SR score. The area under the curve (AUC) was calculated to assess the accuracy of the cut-off, with AUC values of 0.7-0.8, 0.8-0.9, and 0.9-1.0 indicating acceptable, high, and excellent accuracy, respectively (22). The Youden Index was employed to identify the optimal cut-off value, which represent the highest combined sensitivity and specificity. To investigate whether the predictive ability varied, AUC values were computed similarly when focusing exclusively on patients with MDD and when concentrating solely on patients with full-time employment. All data analysis was conducted using SPSS software version 27.0.

## 3 Results

### 3.1 Participant characteristics

A total of 111 patients, with an average age of 43.6 ± 10.3 years, participated in the present study. Out of them, 61 participants were continuously employed during the follow-up period, while the remaining 50 were classified into the non-continuous employment group (**Table 1**). Among the 111 participants, 1 (0.9%) dropped out during the six-month period. This participant was categorized in the non-continuous group due to being on sick leave prior to dropout. The mean QIDS-SR score at baseline was 8.0 ± 4.7 and 11.5 ± 5.5 for the continuous and non-continuous employment groups, respectively. There was no significant difference observed in the demographic characteristics between the two groups, except for the QIDS-SR score.

**Table 1:** Sociodemographic and clinical characteristics.

	Continuous employment (n=61)	Non-continuous employment (n=50)	P-value
Female, n (%)	27 (44.3)	18 (36.0)	0.56
Age, mean ± SD (years)	44.3±10.5	42.7±10.9	0.42
College degrees, n (%)	40 (65.6)	26 (52.0)	0.12
Full-time job, n (%)	42 (68.9)	28 (56.0)	
Psychiatric diagnoses, n (%)			1.00
Major depressive disorder	41 (67.2)	34 (68.0)	
Bipolar disorder	20 (32.8)	16 (32.0)	
QIDS-SR score, mean ± SD	8.0±4.7	11.5±5.5	<.001

QIDS-SR, the 16-Item Quick Inventory of Depressive Symptomatology, Self-Report; SD, standard deviation.

### 3.2 Association of the QIDS-SR score with continuous employment

In the binary logistic regression model, a lower score on the QIDS-SR was linked to an elevated likelihood of continuous employment, presenting adjusted an odds ratio of 1.15 (95% confidence interval (CI): 1.06-1.26, p = 0.001) (**Table 2**). Variables including age, gender, and psychiatric diagnosis were not found to correlate with continuous employment. The Hosmer-Lemeshow test yielded a result of χ² = 6.09 and p = 0.64, demonstrating that the model’s predictions align well statistically with the observed data.

**Table 2:** Correlation of sociodemographic and clinical characteristics with continuous employment.

	β	P-value	OR	95%Cl
Sex	0.29	0.54	1.33	0.53-3.34
Age	-0.02	0.34	0.98	0.94-1.02
Psychiatric diagnosis	-0.20	0.66	0.82	0.33-2.00
The QIDS-SR score	0.14	0.001	1.15	1.06-1.26

OR, odds ration; Cl, confidence interval; QIDS-SR, the 16-Item Quick Inventory of Depressive Symptomatology, Self-Report.

Among the nine individual items, significant differences were observed in weight change, with odds ratios of 0.32 (95%CI: 0.12-0.87, p = 0.025) (**Table 3**). The Hosmer-Lemeshow test yielded a result of χ² = 3.09 and p = 0.93.

**Table 3:** Correlation of sociodemographic and clinical characteristics including the QIDS-SR individual items with continuous employment.

	β	P-value	OR	95%Cl
Sex	0.31	0.56	1.36	0.49-3.75
Age	0.03	0.31	1.03	0.98-1.08
Psychiatric diagnoses	-0.08	0.88	0.93	0.34-2.51
The QIDS-SR individual items				
Sleep disturbance	-0.52	0.37	0.60	0.19-1.86
Depressed mood	-1.24	0.12	0.29	0.06-1.36
Weight change	-1.13	0.03	0.32	0.12-0.87
Concentration loss	-1.02	0.25	0.36	0.06-2.08
Guilty feeling	-0.98	0.12	0.37	0.11-1.28
Suicidal ideation	1.47	0.07	4.33	0.87-21.58
Loss of interest	-0.90	0.19	0.40	0.10-1.57
Fatigue	0.06	0.93	1.06	0.29-3.91
Psychomotor change	0.66	0.35	1.93	0.48-7.70

OR, odds ration; Cl, confidence interval; QIDS-SR, the 16-Item Quick Inventory of Depressive Symptomatology, Self-Report.

### 3.3 Cut-off score of the QIDS-SR score for identifying continuous employment

**Figure 1:**
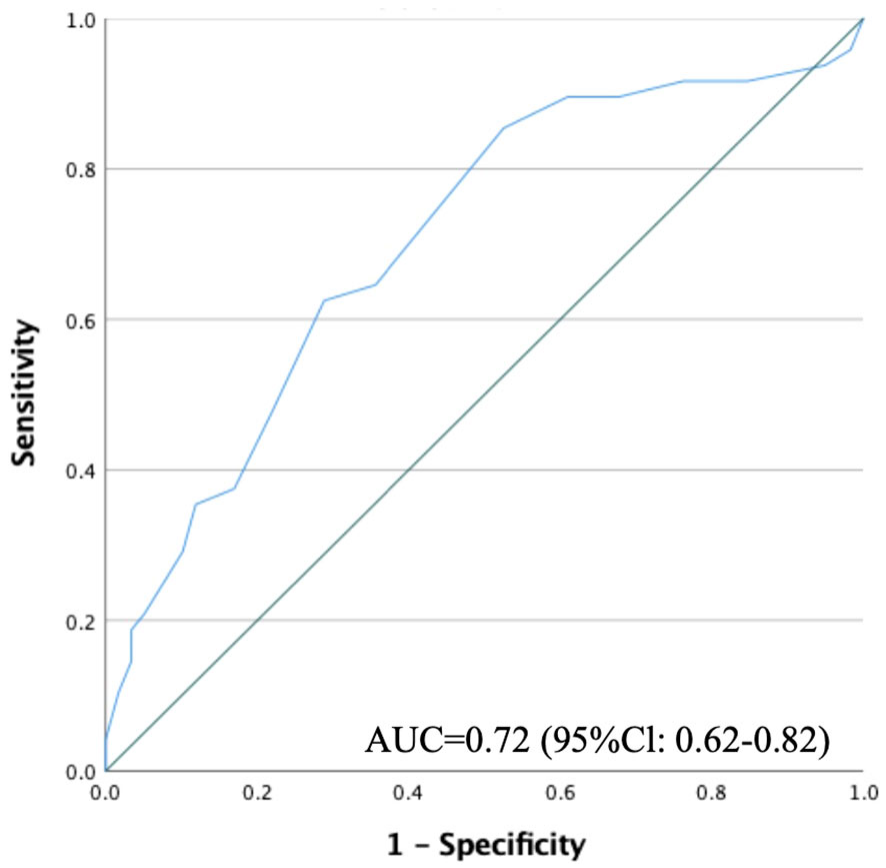
Receiver operating characteristic curve for the QIDS-SR score to predict continuous employment. QIDS-SR, the 16-Item Quick Inventory of Depressive Symptomatology, Self-Report.

The AUC value for identifying continuous employment was 0.70 (95% Cl: 0.60-0.81), indicating acceptable accuracy (**Figure 1**). By using the Youden Index, the optimal cut-off point for identifying continuous employment was determined as a score of 10/11 (**Table 4**). Applying this cut-off yielded sensitivity and specificity values of 63% and 71%, respectively. The positive and negative predictive values were 62% and 68%, respectively.

**Table 4:** The cut-off points of the QIDS-SR score for identifying continuous and non-continuous employment.

Cutoff score	Sensitivity	Specificity	Youden index
9/10	0.65	0.64	0.29
10/11	0.63	0.71	0.34
11/12	0.48	0.78	0.26

QIDS-SR, the 16-Item Quick Inventory of Depressive Symptomatology, Self-Report.

In the subset analysis of participants with MDD, the continuous employment group consisted of 41 patients while the non-continuous group consisted of 34 patients, yielding an AUC value of 0.70 (95% CI: 0.57-0.82). A similar analysis restricted to full-time employment resulted in 42 patients in the continuous group and 28 in the non-continuous group, with an AUC value of 0.77 (95% CI: 0.66-0.89). These focused analyses likewise exhibited acceptable levels of accuracy.

## 4 Discussion

To the best of our knowledge, this study is the first to identify a cut-off value for the QIDS-SR score that predict the likelihood of maintaining employment among outpatients with mood disorders. Our findings indicated an inverse relationship between the baseline QIDS-SR scores and the likelihood of sustained employment. Moreover, we identified a cut-off score of 10/11 for the QIDS-SR to identify continuous employment over six months, with acceptable predictive accuracy. This cut-off score could serve as useful indicators for determining the appropriate work-related course of action for patients with mood disorders.

The findings of this study underscore a notable association between illness severity and absenteeism among individuals with major depression, echoing previous research. Past studies have shown that those on sick leave manifested more pronounced depressive symptoms compared to their actively employed counterparts (16) and symptom severity emerged as a primary driver of absenteeism (17). Notably, while these studies did not delineate specific thresholds predictive of future work discontinuation, our investigation offers an elaborate analysis, examining the impact of individual QIDS-SR scores on employment continuity. Furthermore, weight change was identified as a factor making continued employment challenging in the present study. As patients with residual weight change in remission have been reported to be more prone to relapse (23), this symptom might be linked to maintaining stable social functioning. The present findings can potentially hold significant value in occupational contexts, aiding employers and clinicians in making shared decisions regarding work participation.

The optimal threshold for the QIDS-SR score, indicative of continuous employment over a span of six months, was determined to be 10/11. Intriguingly, this coincides with the recognized demarcation for mild to moderate depression on the QIDS-SR (20). Such a convergence suggests that to ensure uninterrupted work participation, depressive symptoms ought to be curtailed to at least a mild intensity. A prior cross-sectional study assessing disability gradients among 439 patients with major depression revealed pronounced disabilities in those with mild severity compared to their moderately affected counterparts, encompassing absenteeism and general health perception (24). To date, there is a conspicuous lack of standardized criteria or clinical guidelines to assist in determinations regarding recommending sick leaves or reinstating work for affected individuals. Yet, given the predictive insights garnered from our study, symptom alleviation emerges as a crucial determinant in such occupational decisions for those with depressive disorders.

This study presents several limitations that merit attention. Firstly, it is an observational study confined to a 6-month duration. The treatments administered to participants varied throughout the study. Furthermore, depressive symptoms were only assessed at baseline, precluding an analysis of the effects of changes in symptom severity. Secondly, our cohort consisted exclusively of outpatients with depression from Japanese clinical settings. Given the significant disparities in sick leave and unemployment systems across nations, it is imperative to approach the extrapolation of these findings to regions with distinct occupational cultures with caution. Thirdly, the present study is a secondary analysis of a prospective cohort study which focused on assessing the reliability and validity of the Japanese version of the Clinically Useful Depression Outcome Scale Supplemented with Questions for the DSM-5 Anxious Distress Specifier. Given that we utilized the dataset from the original study, power calculations for sample size determination were not conducted for this specific analysis. Fourth, this study included patients who visited Kyorin University Hospital and affiliated hospitals from January 1 to December 31, 2021, representing a consecutive sample. This period corresponds to the increase in remote work during the COVID-19 pandemic. Many individuals might have continued working remotely despite health issues that typically would necessitate sick leave. Fifth, the AUC value obtained in the present study was not optimal. Additionally, focused analyses failed to enhance the predictive value beyond that observed in the total sample. Continuous employment in patients with mood disorders might be influenced by factors beyond illness severity, including comorbid psychiatric or physical disorders, working hours, interpersonal dynamics, and individual work attitudes. Future studies could enhance the prediction of continuous employment by incorporating these additional variables into the analysis. Sixth, our study excluded individuals with suicidal ideation or serious physical illnesses, potentially impacting the findings. As a previous meta-analysis has identified, physical illnesses and greater severity of depression may be as obstacles to re-entering the workforce for people with depression (25).

In summary, our study identified a noteworthy correlation between illness severity, as assessed through the QIDS-SR, and continuous employment among individuals with depression. Consistent employment over a half-year span was predicted by scores below 11 on the QIDS-SR, indicating mild or lower illness severity. This illuminates the importance of measurement-based care and targeted therapy in effectively managing depression among the working population. Moving forward, it would be crucial to further explore these factors through prospective research.

## Data availability statement

The raw data supporting the conclusions of this article will be made available by the authors, without undue reservation.

## Ethics statement

The studies involving humans were approved by the institutional review board at Kyorin University in Tokyo. The studies were conducted in accordance with the local legislation and institutional requirements. The participants provided their written informed consent to participate in this study.

## Author contributions

YM: Conceptualization, Data curation, Formal analysis, Writing – original draft. HS: Conceptualization, Formal analysis, Writing – review & editing. YA: Conceptualization, Writing – review & editing. YT: Conceptualization, Writing – review & editing. IO: Formal analysis, Writing – review & editing. HT: Formal analysis, Writing – review & editing. MM: Data curation, Writing – review & editing. TM: Data curation, Writing – review & editing. TT: Conceptualization, Writing – review & editing. KW: Supervision, Writing – review & editing.
